# Microneedle Vaccination Elicits Superior Protection and Antibody Response over Intranasal Vaccination against Swine-Origin Influenza A (H1N1) in Mice

**DOI:** 10.1371/journal.pone.0130684

**Published:** 2015-06-18

**Authors:** Ju-Hyung Shin, Jae-Keun Park, Dong-Hun Lee, Fu-Shi Quan, Chang-Seon Song, Yeu-Chun Kim

**Affiliations:** 1 Department of Chemical and Biomolecular Engineering, Korea Advanced Institute of Science and Technology (KAIST), Daejeon, Republic of Korea; 2 Avian Disease Laboratory, College of Veterinary Medicine, Konkuk University, Hwayang-dong, Gwangjin-gu, Seoul 143–701, Republic of Korea; 3 Department of Medical Zoology, Kyung Hee University School of Medicine, Seoul, Republic of Korea; University College Cork, IRELAND

## Abstract

Influenza is one of the critical infectious diseases globally and vaccination has been considered as the best way to prevent. In this study, immunogenicity and protection efficacy between intranasal (IN) and microneedle (MN) vaccination was compared using inactivated swine-origin influenza A/H1N1 virus vaccine. Mice were vaccinated by MN or IN administration with 1 μg of inactivated H1N1 virus vaccine. Antigen-specific antibody responses and hemagglutination-inhibition (HI) titers were measured in all immunized sera after immunization. Five weeks after an immunization, a lethal challenge was performed to evaluate the protective efficacy. Furthermore, mice were vaccinated by IN administration with higher dosages (> 1 μg), analyzed in the same manner, and compared with 1 μg-vaccine-coated MN. Significantly higher antigen-specific antibody responses and HI titer were measured in sera in MN group than those in IN group. While 100% protection, slight weight loss, and reduced viral replication were observed in MN group, 0% survival rate were observed in IN group. As vaccine dose for IN vaccination increased, MN-immunized sera showed much higher antigen-specific antibody responses and HI titer than other IN groups. In addition, protective immunity of 1 μg-MN group was similar to those of 20- and 40 μg-IN groups. We conclude that MN vaccination showed more potential immune response and protection than IN vaccination at the same vaccine dosage.

## Introduction

Influenza is one of the most common virus-caused human disease that afflicts the world’s population annually at the scope of regional epidemic and global pandemic. The clinical symptoms of influenza include fever, headaches, fatigue, cough, muscle pain, sore throat, rhinorrhea [1]. About 25–50 million cases of influenza infection occur every year, of which 150,000 hospitalization and 30–40 thousand deaths have been reported in the U.S alone [2]. Among numerous historical cases of influenza outbreaks, the 2009 new swine-origin H1N1 influenza was the first pandemic influenza to occur in the 21^st^ century. Since the initial outbreak from Mexico, the disease rampantly swept across the world, inflicting hundreds of thousands of human infection, hospitalization and death cases in over 200 nations [3]. In addition, the recent outbreak of a new H7N9 influenza virus strain in China, which has inflicted 123 infection cases and 37 deaths, has rung an international alarm in the healthcare industry over the preparedness against such rapidly and dangerously evolving influenza virus strains [4]. In order to prevent the occurrence of a pandemic influenza, vaccination has been proposed as one of the most promising tools to control the infection of the virus in humans [5, 6].

The conventional vaccine delivery tool to administer pharmaceutical formulations into a patient’s muscle (intramuscular) or subcutaneous tissue (subcutaneous) is the hypodermic needle. However, there are lots of limitations associated with this technique. Firstly, there are several needle-related safety issues such as needle stick injury and blood-borne infection through reused needles in developing countries. Furthermore, the use of an invasive needle can cause a decline in patient compliance due to needle phobia, stress and pain, and the need of properly trained health-care personnel for vaccination [7, 8]. Thus, to counter these disadvantages, novel vaccine administration tools such as intranasal injection, microneedle patch, jet injector and tattoo vaccination method have been suggested to replace the conventional intramuscular or subcutaneous injection, as showing their own advantages, such as minimally invasive injection with negligible pain [9–12].

One such novel delivery tool, microneedles (MN, S1 Fig), is an array of micron-sized needles which penetrate across the skin barrier, the *stratum corneum*, and deliver therapeutic materials into the underlying immune cell-rich skin regions, the epidermis and dermis [13]. Unlike conventional intramuscular vaccination done by a hypodermic syringe, which causes pain and muscle aches at the injection site [14, 15], the minuscule needle dimensions of MN minimizes any pain with remarkable improvement in patient compliance [13, 16, 17]. There are hosts of influenza vaccine antigen that are being investigated with MN, including inactivated influenza virus [16], influenza virus-like particle [18], recombinant trimeric soluble influenza hemagglutinin [19], and hemagglutinin DNA [20].

Another potential tool for substituting traditional vaccinations is the mucosal-stimulating method. Mucosal immunizations span the spectrum of oral, intranasal, pulmonary, ocular, vaginal, and rectal routes [12], and focus on the targeting of mucosal tissue, which is one of immune systems in human body (including the gastrointestinal tract, the upper and lower respiratory tract, and the urogenital tract) [21]. Among these systems, intranasal (IN) vaccination, by way of administering drugs into the nostril, elicits the most notable level of systemic and mucosal immune responses in the nasal epithelium and nasal-associated lymphoid tissue (NALT) of the lungs and the upper respiratory tract [10, 11, 22]. Furthermore, IN vaccination is a needle-free injection, so it is impossible to infect patients with any blood-borne disease or cause needle phobia [10]. Furthermore, it is also easy to deliver the desired drugs into the mucosal tissue to vaccinate mass populations against air-borne pathogens [12, 22]. This tool is currently commercialized by MedImmune and Serum Institute of India [10]. For example, FluMist, manufactured by MedImmune and approved for human use in 2003, consists of live attenuated trivalent influenza viruses (H1N1, H3N2 subtypes, and B) [23, 24].

To the best of our knowledge, there has been no report comparing the immunogenicity and protection efficacy against influenza virus infection in the mouse model. Therefore, in this study, we demonstrated which administration route elicited better immune response and protection against homogeneous challenge using inactivated 2009 A/H1N1 and dose-sparing effect by measuring the total level of antigen-specific immunoglobulin G (IgG), IgG subtypes, and hemagglutination-inhibition (HI) activity in the mouse sera and lung viral titer after challenge, and by monitoring the mouse conditions against lethal challenge.

## Materials and Methods

### Ethics statement

All animal procedures performed in this study (permit number:KU14123) were reviewed, approved, and supervised by the Institutional Animal Care and Use Committee of Konkuk University.

### Preparation of inactivated viruses

A/California/07/09 (H1N1) (kindly provided by Korea Centers for Disease Control and Prevention) was propagated in the allantoic cavities of 9–11 day old specific pathogen free (SPF) embryonated chicken eggs (ECE). Seventy-two hours after inoculation at 37°C, allantoic fluid containing H1N1 virus was collected and clarified by low speed centrifugation (2,000 × g, 30 min, 4°C), and chemically treated with formalin (final concentration of 0.2%) for 24 h at 22°C for virus inactivation. Formalin-treated allantoic fluid was stored at 4°C until the confirmation of virus inactivation. The inactivation of virus was confirmed by the inoculation of 0.2 ml of formalin-treated allantoic fluid into five 10 days-old-embryonic eggs. After 72 h of incubation at 37°C, the allantoic fluids from all ECEs showed negative results for hemagglutination activity with chicken red blood cell (RBC). After the confirmation of inactivation, the inactivated H1N1 virus from the clarified supernatants was pelleted (30,000 × g, 1.5 h, 4°C). The pelleted virus was resuspended in phosphate-buffered saline (PBS) solution (pH 7.4) and purified using 20–50% (w/v) discontinuous sucrose density gradient purification (150,000 × g, 2.5 h, 4°C). The protein concentration of purified inactivated virus was determined by QuantiPro Bicinchoninic Acid (BCA) Assay kit (Sigma) according to the manufacturer's instructions.

### Fabrication and coating of MN

All steps were conducted as in S2 Fig. Stainless steel in-line microneedles (S1 Fig. Tech-Etch, Plymouth, MA) were dipped into a modified coating device containing the coating solution, previously described [25] and dried at room temperature for 1 day. The coating solution used for the dipping process consisted of 1.0% (w/v) Carboxymethylcellulose sodium salt (CMC, Sigma, St. Louis, MO), 0.5% (w/v) Lutrol F68 (Sigma), 15% (w/v) D-(+)-Trehalose dihydrate (Sigma) and 3 mg/ml inactivated H1N1 virus in PBS by QuantiPro BCA Assay kit.

### Preparation of IN vaccination

In order to intranasally deliver the same quality and amount of vaccine as MN vaccination, vaccine-coated MN were fabricated and dried at room temperature for 1 day, as described above. After drying, in order to completely dissolve virus from the MN, vaccine-coated MN were incubated in PBS solution (50 μl/array) for 12 hours at 4°C. Dissolved antigen stored at 4°C was shortly used for IN immunization within 2 hours, as described by Quan *et al* [26]. PBS containing dissolved virus was directly used for intranasal inoculation.

### Quantitative analysis of protein on coated MN and virus solution

The coated MN made by the previous procedure were dissolved in 200 μl of PBS solution and incubated at 4°C for 12 hours. Then, the amount of proteins coated on the MN and the concentrations of vaccine solutions were measured by QuantiPro BCA Assay kit. The protein concentration coated on the MN was approximately 1 μg.

### Immunization and challenge

Forty-four six-week BALB/c mice (Orient Bio, Sungnam, Korea) were prepared before immunization and classified into 4 groups: eleven mice which were immunized by MN vaccination with inactivated H1N1 virus (MN group), eleven mice which were immunized by IN vaccination with inactivated H1N1 virus (IN group), eleven mice which were not treated by any immunization, but were challenged (Naïve group), and eleven mice which were not treated by any immunization and were excepted from challenge (Control group). Mice in the MN group and IN group were anesthetized with Avertin (375 mg/kg) intraperitoneally. During anaesthesia, the fur on all murine back in the MN group was removed with a depilatory cream (Veet, Reckitt Benckiser, Berkshire, UK) and washed by using warm water and soaked cotton ball (70% ethanol) after application of the removal cream for 5 min.

After drying by a hairdryer, the coated MN were manually inserted into the site where the hair was removed off, left for 10 min to allow the complete dissolution of all coating solution into the skin of mouse, and picked out. One microneedle array was used to deliver 1 μg of inactivated H1N1 antigen for each mouse At the same time, the mice in the IN group were intranasally injected with 1 μg of inactivated H1N1 dissolved in 50 μl of PBS by nasal dripping, previously prepared. The mice in the Naïve group and the Control group were not treated by any immunization tool.

After 5 weeks, the mice were anesthetized by intraperitoneal injection of Avertin (375 mg/kg) and challenged intranasally with 90 μl of 10^6.0^ EID_50_ A/Korea/01/09 (H1N1) (kindly provided by Korea Centers for Disease Control and Prevention) for the challenge experiment. At 4 days post-challenge, four mice from each group except the control group were sacrificed, and the lungs were collected for determining the lung virus titers in the infected mice. The remaining mice were observed and their weight and survival rate were recorded for 14 days after challenge. Mice which lost over 25% of their weight were considered dead and humanly euthanized. In this study, the euthanasia was performed by cervical dislocation under anesthetic condition using intraperitoneal injection of Avertin (375 mg/kg).

### Measuring antibody response and HI titer

Antibody responses (total IgG, IgG1, IgG2a) in mouse sera collected at 2 and 4 weeks after immunization were measured using enzyme-linked immunosorbent assay (ELISA). Inactivated antigen used for the immunization were diluted with PBS (2 μg/ml), added into 96-well plates (50 μl/well), and incubated overnight at 4°C. After the plates were washed, wells were blocked by 5% skim milk (Sigma, St. Louis, MO) and incubated at 37°C for 1 hour. After the plates were washed with PBS containing 0.05% Tween 20 (PBST, Samchun Chemical, Korea), mouse sera which were two-fold diluted into 2.5% skim milk suspended in PBST were transferred into the plates. After incubation (20°C, 90 min) and another washing session, HRP (Horseradish peroxidase)-conjugated anti-mouse IgG, G1, or G2a antibody (AbDSerotec, UK) was added. Then, TMB (3, 3′, 5, 5′-Tetramethylbenzidine) buffer (SurModics, MN, USA) was added to wells (100 μl/well), and 1N hydrochloric acid (100 μl/well) was used for stopping the reaction. After all the steps were finished, the plates were measured in an ELISA reader at 450nm for determining the optical density (OD).

To determine HI titers, HI assay were conducted as previously described [27]. Serum samples were first treated with a receptor-destroying enzyme (Denka Seiken, Tokyo, Japan) by incubation overnight at 37°C and then for 30 min at 56°C. Sera were serially diluted, mixed with 4 haemagglutination (HA) units of H1N1 virus, and incubated for 30 min at room temperature prior to adding 0.5% chicken red blood cells. The reciprocal of highest serum dilution preventing hemagglutination was scored as the HI titer.

### Measuring lung virus titer

In order to determine the lung virus titer, at day 4 post-challenge, the lungs were collected and homogenized in PBS 10% (w/v). The homogenates were centrifuged at 1400 × g for 10 min at 4°C. The infectivity of the virus in the supernatant was determined with a plaque assay using Madin–Darby canine kidney (MDCK) cells [28].

### Injecting different dosages for IN vaccination

To confirm the immunogenicity of vaccine and how much dosage would be needed for similar immune response and protection effect to MN with 1 μg, escalating doses of inactivated H1N1 antigen (1, 10, 20, and 40 μg), the same antigen coated on MN, was intranasally delivered, and the immunogenicity and protective efficacy were compared with 1 μg of MN vaccination. A solution consisting of 1.0% (w/v) CMC, 0.5% (w/v) Lutrol F68, 15% (w/v) D-(+)-Trehalose dihydrate and 3 mg/ml inactivated H1N1 virus, which consist of the same composition used in the previous experiment, was spread and dried at RT for 1 day on sheets of stainless steel, which is the same type of stainless steel as MN, to mimic storing conditions of coated virus on MN, as previously described [16, 27]. The amount of virus spread on sheets was controlled by releasing the volume of coating solution. After drying for 1 day, vaccine-coated stainless steel sheets were incubated into PBS solution (100 μl/sheet) at 4°C for 12 hours to dissolve the vaccine from the sheets. Dissolved antigen stored at 4°C was shortly used for IN immunization within 2 hours, based on modified quantitation method described by Quan et al [26]. PBS containing dissolved virus was directly used for intranasal inoculation. Twenty-five six-week BALB/c mice (Orient Bio) were prepared for this experiment. All mice except for Naïve groups anesthetized by Avertin (375 mg/kg) were intranasally injected with 100 μl of inactivated virus solution. Each IN group (5 mice per a group), was intranasally immunized with 1, 10, 20, or 40 μg of inactivated H1N1 virus dissolved in PBS. Mice sera were collected at 2 and 4 weeks post immunization to measure the antibody titer (total IgG, IgG1 and IgG2a) and HI titer. After 5 weeks post immunization, all mice which were anesthesized with Avertin (375 mg/kg), and challenged with 90 μl of 10^6.0^ EID_50_ of H1N1 virus, the same challenge condition used for MN group. Mice were observed daily for 14 days to measure the weight loss and survival rate. The immunogenicity and protective efficacy of IN immunization with escalating doses of antigen were compared to that of 1 μg MN immunization, which has been previously conducted in this study. Experiment for the MN group was conducted independently in a separate challenge experiment using the same lethal dosage.

### Statistical analysis

Every assay was measured using at least three replicate samples, from which the arithmetic mean and standard error of the mean were calculated (unless otherwise noted). A two-tailed Student's t-test was performed when comparing two different conditions. When comparing three or more conditions, a one-way analysis of variance (ANOVA; α = 0.05) was performed. A p-value less than 0.05 was considered to be significant.

## Results

### Antibody responses to MN and IN vaccination

**Fig 1:**
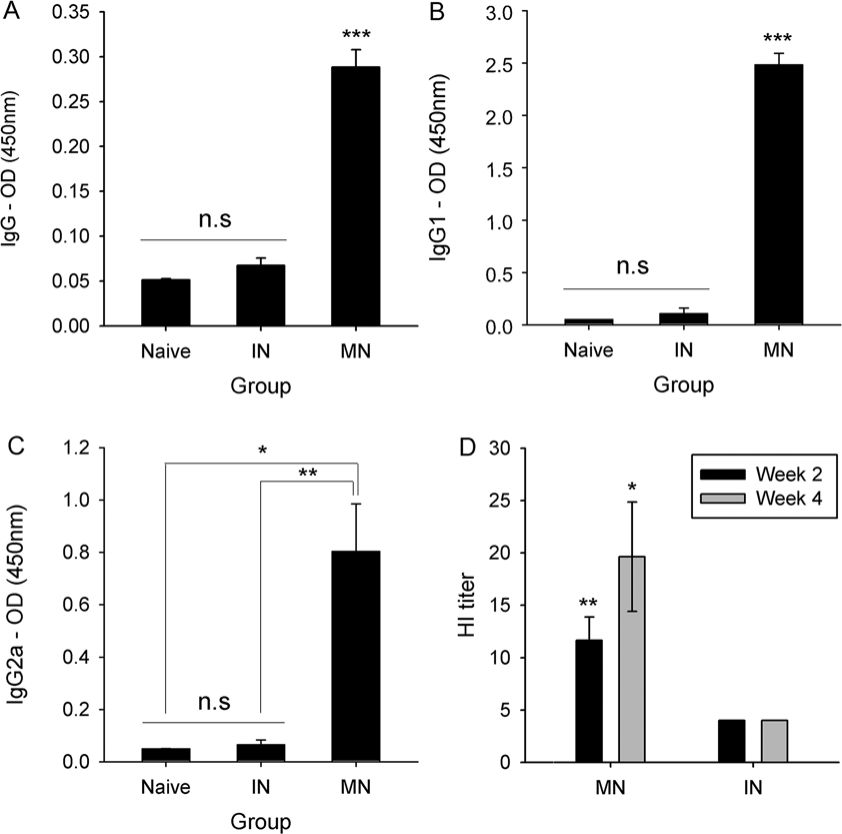
Antigen-specific antibody responses and HI titer in MN or IN-immunized sera with 1 μg. Antigen-specific antibody responses at week 4 (IgG—A, IgG1—B and IgG2a—C) and HI titer (D) at week 2 and week 4 in mice sera immunized by IN or MN vaccination. Mice sera injected by MN or IN immunization were collected biweekly and measured for their HI titer and IgG, IgG1 and IgG2a levels by HI assay and ELISA. n = 11 for HI titer and n = 5 for antibody titer. Error bars represent standard errors. *, p<0.05; **, p<0.005; ***, p<0.001. n.s, not significant.

To compare the immunogenicity of the two vaccine delivery methods, 1 μg of inactivated H1N1 virus was administered into mice by either MN or IN injection. Immunoglobulin G (IgG) and IgG subtype (IgG1 and IgG2a) antibody responses were measured by ELISA from the sera collected 4 weeks after immunization. As shown in Fig 1A, the mean serum IgG level in the MN group was 0.29. The IgG level in the IN group was 0.07, which was significantly lower than that of the MN group and similar to that in the Naïve group (p<0.001). In addition, the IgG1 and IgG2a level (Fig 1B and 1C) were significantly higher in sera from MN-immunized mice than in sera from IN-immunized mice (p<0.001). However, the antibody isotype titers in sera from IN-immunized mice were similar to those seen in the Naïve group (p = 0.928 for IgG1 and p = 0.997 for IgG2a).

### HI assay

In addition to the antibody response against influenza virus, immunogenicity was evaluated by the HI assay performed on immunized sera collected from each study group at weeks 2 and 4 (Fig 1D). In the MN group, the average HI titer was 12 at week 2 and 20 at week 4. However, in the IN and Naïve groups, the HI titer did not change following immunization, indicating the effectiveness of MN vaccination on inducing HI antibodies against influenza virus.

### Protective immunity

**Fig 2:**
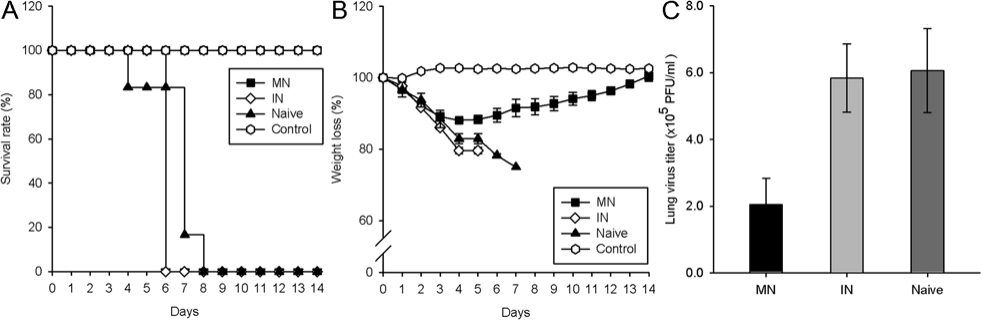
Protection effect against lethal dosage challenge. Survival rate (A), weight loss (B) were measured for 14 days (n = 7). At day 4 post-challenge, lungs were collected and lung virus titer (C) were measured by plaque assay of MDCK cells (n = 4). Error bars represent standard errors. *, p<0.05; **, p<0.005; ***, p<0.001. n.s, not significant.

A lethal homologous challenge was performed to compare the protective efficacy of the vaccination methods. At 14 days, the survival rate was 100% in the MN (7/7) (Fig 2A). However, the challenge was lethal in the IN (0/7) and Naïve groups (0/6), in which the mice had to be euthanized on days 6 and 8, respectively. Mice in the MN group had lost about 12% of their initial weight by day 4, but gradually regained their initial weight between day 4 (88.1%) and day 14 (100.5%) (Fig 2B). By day 5, mice in both the IN and Naïve groups had lost over 25% of their body weight. For additional confirmation of the difference in protection conferred by MN and IN injection, lung viral titers were measured by the plaque assay in MDCK cells (Fig 2C). A difference (p = 0.055) in the extent of viral replication was observed in the MN group (2.0510^5^ PFU/ml) and the IN group (5.8410^5^ PFU/ml). However, similar titers were found the IN and Naïve groups (6.0610^5^ PFU/ml, p = 0.987). Therefore, MN coated with inactivated influenza virus showed better protective immunity in mice against lethal challenge than IN vaccination.

### Comparing escalating dosages for IN vaccination with MN vaccination with 1 μg dose

We tried to confirm the immunogenicity of antigen using in IN immunization and determine the IN dose that would induce immune responses equivalent to those after MN administration because MN administration of the influenza vaccine was more immunogenic and conferred better protective immunity than IN administration at the 1 μg of dosage. Therefore, 1, 10, 20, or 40 μg of inactivated H1N1 virus was intranasally administered to mice.

**Fig 3:**
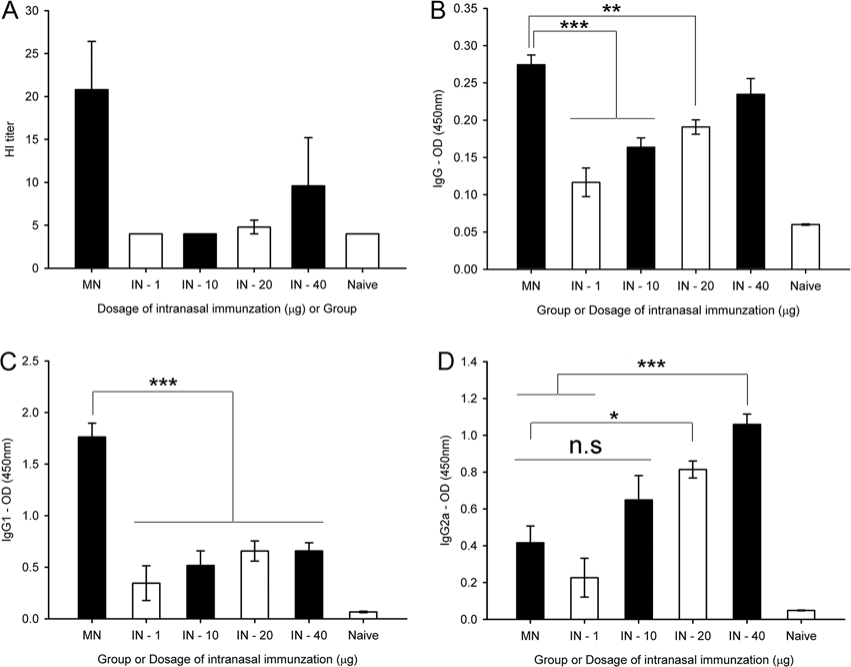
Antigen-specific antibody responses and HI titer in IN-immunized sera with 1, 10, 20 and 40 03BCg. HI titer (A) and antigen-specific antibodies (IgG—B, IgG1 – C and IgG2a—D) responses at week 4 in mice sera immunized by IN or MN vaccination. In the case of the IN vaccination group, mice were immunized by administering 1, 10, 20, 40 μg of inactivated H1N1 virus in 100μl of PBS solution to each group. Mice sera were measured for HI titer and IgG, IgG1 and IgG2a levels by HI assay and ELISA, respectively. Mice sera immunized by MN vaccination were collected after immunization. In the case of HI assay, the HI titer for MN was measured independently. n = 5 for HI titer and antibody titer. Error bars represent standard errors. *, p<0.05; **, p<0.005; ***, p<0.001. n.s, not significant.

The HI activity was lower at all IN doses than that observed after immunization with 1 μg of MN vaccine (Fig 3A). Mice immunized intranasally with either 1 or 10 μg of inactivated H1N1 virus did not have detectable HI activity. In addition, the HI titer was much higher in mice given 1 μg of the MN vaccination than in those injected intranasally with doses of 20 and 40 μg, which resulted in HI titers of 4.8 and 9.6, respectively. MN-immunized mice also had higher antigen-specific IgG and IgG1 levels than mice given IN-administered doses of 1, 10, or 20 μg of vaccine (Fig 3B and 3C; p<0.001—MN:IN-1, MN:IN-10 in IgG, MN:IN-1, MN:IN-10, MN-IN-20 in IgG1; p<0.005 – MN:IN-20 in IgG). However, the IgG2a response in MN-immunized sera was similar to that of IN-immunized sera after administration of 1 or 10 μg of vaccine, but was significantly lower than in those given 20 (p<0.05) or 40 μg (p<0.001) of vaccine.

The much higher antigen-specific IgG and IgG1 levels observed in mice immunized using MN coated with 1 μg of vaccine than in mice immunized with 40 μg of IN vaccine offers strong evidence that antigen by MN vaccination is more efficient than that by IN vaccination (Fig 3D).

### Dose-dependent protective immunity of IN immunization

**Fig 4:**
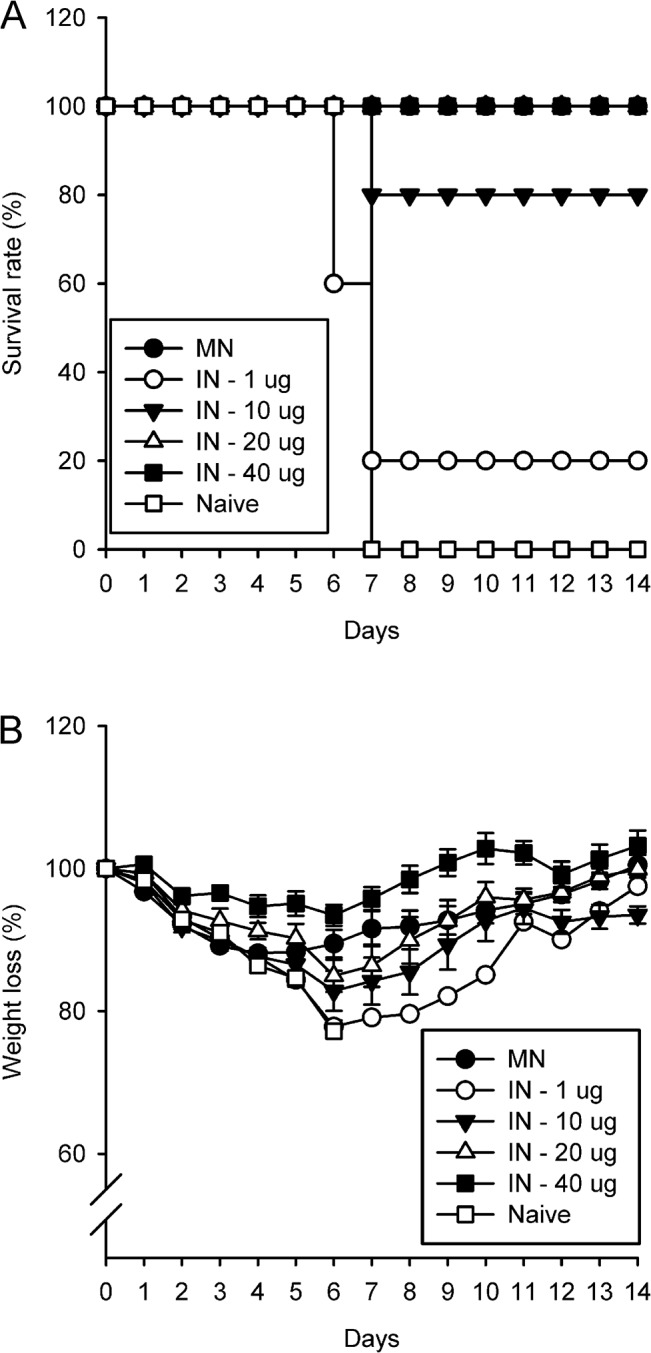
Dose-dependent protection against lethal challenge. Survival rate (A) and weight loss (B) were observed for 14 days after challenge in all intranasally immunized mice injected with various dosages (IN—1, 10, 20, 40 μg, each) and mice in the Naïve group which were not immunized. Experiment for the MN group was conducted independently in a separate challenge experiment using the same lethal dosage. Error bars represent standard errors. n = 5, each.

At 4 weeks post immunization, mice in the Naïve group and in the IN vaccination group inoculated with 1, 10, 20, or 40 μg of inactivated H1N1 virus lethally challenged with H1N1 virus. During the 14-day post-challenge period, all mice immunized with 20 μg (5/5) or 40 μg (5/5) of vaccine survived the lethal challenge (Fig 4A), whereas those vaccinated with 10 μg of vaccine showed partial protection (4/5) and those vaccinated with 1 μg clearly showed an sub-protective immunological response, as shown by the survival of only one of the five mice. The dose-dependent trend of protection can be clearly seen in Fig 4B. Mice administered 1 μg of vaccine intranasally had the highest percentage of initial body weight loss at day 6 (22.2%), and the weight loss steadily declined with increases in the amount of vaccine (10 μg, 17.2%; 20 μg, 15.0%; 40 μg, 6.5%). However, the IN group mice immunized with 20 μg vaccine showed a similar tendency as the MN group. Overall, IN immunization resulted in a dose-dependent increase in protection against an H1N1 influenza challenge.

## Discussion

Vaccination plays a key role in preventing the spread of highly infectious diseases, such as in the early stage of influenza infection. However, in the case of influenza, vaccine production is currently limited by delays in production and inadequate output from production facilities. These limitations prevent the control of infection during the early stage, before the affection spreads to the entire population and becomes a seasonal epidemic or pandemic [29–31]. Therefore, novel vaccine delivery devices have been proposed to improve vaccine efficiency and patient compliance, and reduce the required dosage and costs [8]. For example, needle-free devices such as jet injection and powder injection have been introduced. Currently, a wide range of vaccine delivery devices with different routes of administration are available. These include transdermal, oral, sublingual, nasal and pulmonary immunization techniques [7, 9]. Transdermal vaccine delivery, which has received significant attention, depends on the presence of a relatively large population of immune cells, such as Langerhans cells, dermal dendritic cells and CD8+ T cells, in the epidermis and dermis of the skin [32, 33]. Other methods, such as oral, sublingual, nasal and pulmonary delivery, target the gastrointestinal or respiratory mucosa and mucosa-associated lymphoid tissue [34]. In the current study, we compared the immunogenicity and protection afforded by two promising vaccine delivery tools against the 2009 swine-origin influenza A (H1N1) virus in a mouse model. MN is a transdermal delivery system, and IN delivery targets the nasal-associated lymphoid tissue. This is the first study showing the superiority of MN vaccination over IN vaccination. Previous studies compared MN and intramuscular vaccination [16, 24, 35], but to our knowledge, no study has compared directly IN and MN vaccination. In the present study, to make it easier to compare the immunological features of vaccines delivered by different routes, we used an inactivated influenza virus that was previously used as the standard antigen [16, 36], although a live attenuated influenza virus vaccine is known to give better protection than IN vaccines [37].

Antibody responses and HI activity after immunization are key criteria for measuring vaccine immunogenicity. In this study, mice immunized with influenza vaccine-coated MN showed antigen-specific immune responses and HI titers that were consistent with those reported previously [27, 38]. However, in contrast to MN vaccination, IN vaccination with 1 μg of inactivated H1N1 virus failed to elicit measurable immune responses, i.e., HI activity and antibody titers were similar to those in the Naïve group. Mice receiving the IN vaccine all died after a virus challenge, but all the mice in the MN group survived. In addition, MN vaccination resulted in better reduced viral replication from the lungs than IN vaccination, as observed previously [38, 39]. It can be assumed that the vaccine doses administered intranasally were insufficient to induce an adequate immune response in the mice [22]. Consistent with our findings, Ichinohe *et al*. [40] found that a low IN dose (1 μg) of trivalent influenza vaccine does not induce a protective immune response in mice. Moreover, 100 μg of inactivated influenza virus is needed to elicit a sufficient heterosubtypic immune response when given intranasally [36]. Although it is not appropriate to compare the specific amount of antigen using in these studies because there are several different assays using for protein quantification, above data could be used as a rough indicator of dosage and trends for intranasal vaccination.

To determine whether the vaccine used in that experiment could elicited immune response and the amount of vaccine requested for similar protection effect of 1 μg of vaccine-coated MN, mice were intranasally administered with 1, 10, 20, or 40 μg of inactivated H1N1 viruses, and the subsequent results on the immunogenicity and protective efficacy of IN vaccination were compared to those of 1 μg MN vaccination. The virus was the same as that used in the previous experiment, but more virus and a larger volume (100 μl) were used in this study because poor antibody responses and lack of protective immunity are often associated with insufficient doses of vaccine [41]. According to the reports by Ichinohe *et al*. [40] and Takada *et al*. [36], although the amount of antigen used in both studies were not described in details, it could be inferred that higher vaccine doses are likely to be needed for IN than for MN immunization in mice to induce a detectable level of immune response or HI activity.

So, HI titers, and total IgG and IgG1 responses showed that much higher dosages were required in the IN group to achieve immune responses such as those in the MN group. Specifically, the MN vaccination resulted in a considerably higher IgG response when compared with IN vaccination (Fig 1B). In line with this result, a dose-dependent protection effect was observed after virus challenge, such that a 100% survival rate and only a slight weight loss were observed with 20 μg and 40 μg doses of IN vaccine; thus, protection effect induced by MN vaccination with 1 μg was as similar to that induced by IN vaccination with 40 μg doses of IN vaccine. The volume of antigen injection via IN route used in this study might have caused the delivery of inactivated antigen to the pulmonary region. However, the volumes previously used for the IN delivery of antigen in mice are variable from different studies (e.g. 2ul [42], 10ul [43], 20ul [44], 30ul [45], 50ul [46]). Further study on optimization of injection volume of IN delivery of inactivated antigen used in this study would be needed, however it is clear that the protective efficacy induced by MN vaccination with 1 μg of inactivated swine A/H1N1 virus was greater than that induced by IN vaccination with the same amount of antigen. Although the chemicals including in coating formulation of MN and IN, such as CMC and Lutrol F68, have mucoadhesive characteristics and affect mucus rheology and subsequent vaccine efficacy [47–54], the amount of each chemical delivered into nostril was very small (CMC: 0.01~0.27%, Lutrol F68: 0.01~0.13%) and much lower than previous studies (CMC: 0.1~2.5%, Lutrol F68: 0.3~20%). So, the coating formulation used in this study would not affect the immune responses after IN immunization. Overall, the immune response to IN vaccination was dose-dependent, whereas MN vaccination was superior to IN vaccination, based on the amount of dosage.

As mentioned previously, the skin is a strongly immunogenic organ due to the abundance of antigen-presenting cells such as Langerhans cells and dermal dendritic cells [33]. Delivery of vaccine directly into such an immune cell-rich organ using microneedles brings about improved immune responses and a dose-sparing effect, comparing with IM [39]. Microneedle-based skin vaccination technology reduces the amount of antigen required for vaccination, which results in reduced vaccination cost and overcomes vaccine shortage problems [39]. Moreover, as the cost is competitive with conventional hypodermic syringes and needles due to small package volume for logistic process, requiring relatively small number of healthcare personnel, low material costs [55], microneedles may serve as an effective vaccination tool for mass vaccination during pandemics or seasonal epidemics [56].

In this study, MN vaccination was superior to IN vaccination at the same dose of inactivated 2009 swine-origin influenza A (H1N1) virus, and showed similar protection effect to IN vaccination with 20-fold higher dosage. Previous preclinical [39] and clinical [56] studies have shown that MN vaccination can achieve high level of immune response with much lower vaccine doses than that required by intramuscular vaccination. One notable study of heterosubtypic cross-protection reported similar trends in antibody levels and other immunogenicity features following subcutaneous, IN, and intramuscular administration [57]. However, no previous studies have compared the immunological responses of IN and MN vaccination. MN vaccination is a promising vaccine delivery tool that might supplant traditional vaccination methods in the near future.

## Conclusion

In this study, the immunogenicity and protective efficacy of IN and MN vaccination against the 2009 swine-origin influenza A (H1N1) virus were compared in a mouse model. The same dosage of inactivated H1N1 virus was administered by the IN route or into the skin by MN injection. MN-vaccinated mice showed higher antigen-specific antibody responses, higher HI activity, better protection, and decreased viral replication following a lethal challenge than IN-vaccinated mice. In following experiment, immune responses to IN vaccination with escalating doses of antigen were compared with those induced by MN vaccination. MN vaccination, even with 1 μg of antigen, provided similar level of antibody responses (IgG and IgG1) and immunological protection as those of IN vaccination with 40 μg of antigen, demonstrating that the MN vaccination showed similar protection effect to IN vaccination with 40-fold higher dose. The positive features of microneedle delivery, including improved patient compliance, reduced anxiety and pain, possibility of self-administration, and ease of transportation, in addition to the superior efficiency confirmed in this study, demonstrate that MN vaccination is a promising vaccine delivery route suitable for mass vaccination campaigns during seasonal epidemics or pandemics [13, 39]. In conclusion, MN vaccination provides a significant immune response and protection effect compared with IN vaccination for the 2009 swine-origin influenza A virus vaccine.

## Supporting Information

S1 Fig Microneedles coated with bovine serum albumin (left) or Sulforhodamine B (right). In-line stainless steel microneedle array was coated with coating solution plus BSA or Sulforhodamine B and observed by optical microscopy or fluorescence microscopy, respectively. Scale bar = 100 μm.
(TIF)

S2 Fig Experimental design for comparison study between microneedle and intranasal immunization. One group of mice were immunized with a vaccine solution, consisting of 1 μg of inactivated swine H1N1 virus vaccine constituted in a coating solution, by injection of the solution into nostrils. In the other group of mice, vaccine-coated MN were inserted into the back skin of mice, which were coated with the same dosage of the vaccine as in IN vaccination.
(TIF)

## References

[1] Holmgren J, Svennerholm AM. Vaccines against mucosal infections. Curr Opin Immunol. 2012;24(3):343–53. 10.1016/j.coi.2012.03.014 . 22580196

[2] Girard MP, Cherian T, Pervikov Y, Kieny MP. A review of vaccine research and development: human acute respiratory infections. Vaccine. 2005;23(50):5708–24. 10.1016/j.vaccine.2005.07.046 . 16154667 PMC7130922

[3] Girard MP, Tam JS, Assossou OM, Kieny MP. The 2009 A (H1N1) influenza virus pandemic: A review. Vaccine. 2010;28(31):4895–902. 10.1016/j.vaccine.2010.05.031 . 20553769

[4] Yu H, Cowling BJ, Feng L, Lau EH, Liao Q, Tsang TK, et al Human infection with avian influenza A H7N9 virus: an assessment of clinical severity. The Lancet. 2013;382(9887):138–45. 10.1016/S0140-6736(13)61207-6 23803487 PMC3801178

[5] Palese P. Influenza: old and new threats. Nat Med. 2004;10:S82–S7. 15577936 10.1038/nm1141

[6] Osterholm MT, Kelley NS, Sommer A, Belongia EA. Efficacy and effectiveness of influenza vaccines: a systematic review and meta-analysis. Lancet Infect Dis. 2012;12(1):36–44. 10.1016/s1473-3099(11)70295-x . 22032844

[7] Amorij J-P, Kersten GF, Saluja V, Tonnis WF, Hinrichs WL, Slütter B, et al Towards tailored vaccine delivery: Needs, challenges and perspectives. J Controlled Release. 2012;161(2):363–76. 10.1016/j.jconrel.2011.12.039 22245687

[8] Giudice EL, Campbell JD. Needle-free vaccine delivery. Adv Drug Del Rev. 2006;58(1):68–89. 16564111 10.1016/j.addr.2005.12.003

[9] Kim YC, Jarrahian C, Zehrung D, Mitragotri S, Prausnitz MR. Delivery systems for intradermal vaccination. Curr Top Microbiol Immunol. 2012;351:77–112. 10.1007/82_2011_123 21472533 PMC3173582

[10] Lycke N. Recent progress in mucosal vaccine development: potential and limitations. Nat Rev Immunol. 2012;12(8):592–605. 10.1038/nri3251 . 22828912

[11] Amorij JP, Hinrichs WLJ, Frijlink HW, Wilschut JC, Huckriede A. Needle-free influenza vaccination. Lancet Infect Dis. 2010;10(10):699–711. 10.1016/s1473-3099(10)70157-2 . 20883966

[12] Mitragotri S. Immunization without needles. Nat Rev Immunol. 2005;5(12):905–16. 10.1038/nri1728 . 16239901

[13] Kim YC, Park JH, Prausnitz MR. Microneedles for drug and vaccine delivery. Adv Drug Deliv Rev. 2012. 10.1016/j.addr.2012.04.005 22575858 PMC3419303

[14] Clark TW, Pareek M, Hoschler K, Dillon H, Nicholson KG, Groth N, et al Trial of 2009 influenza A (H1N1) monovalent MF59-adjuvanted vaccine. New Engl J Med. 2009;361(25):2424–35. 10.1056/NEJMoa0907650 19745215

[15] Plennevaux E, Sheldon E, Blatter M, Reeves-Hoché MK, Denis M. Immune response after a single vaccination against 2009 influenza A H1N1 in USA: a preliminary report of two randomised controlled phase 2 trials. The Lancet. 2010;375(9708):41–8. 10.1016/S0140-6736(09)62026-2 20018365

[16] Kim YC, Quan FS, Compans RW, Kang SM, Prausnitz MR. Formulation and coating of microneedles with inactivated influenza virus to improve vaccine stability and immunogenicity. J Control Release. 2010;142(2):187–95. 10.1016/j.jconrel.2009.10.013 19840825 PMC2823933

[17] Gill HS, Denson DD, Burris BA, Prausnitz MR. Effect of microneedle design on pain in human subjects. The Clinical journal of pain. 2008;24(7):585 10.1097/AJP.0b013e31816778f9 18716497 PMC2917250

[18] Quan FS, Kim YC, Vunnava A, Yoo DG, Song JM, Prausnitz MR, et al Intradermal Vaccination with Influenza Virus-Like Particles by Using Microneedles Induces Protection Superior to That with Intramuscular Immunization. J Virol. 2010;84(15):7760–9. 10.1128/jvi.01849-09 . 20484519 PMC2897640

[19] Weldon WC, Martin MP, Zarnitsyn V, Wang B, Koutsonanos D, Skountzou I, et al Microneedle vaccination with stabilized recombinant influenza virus hemagglutinin induces improved protective immunity. Clin Vaccine Immunol. 2011;18(4):647–54. 10.1128/CVI.00435-10 21288996 PMC3122571

[20] Song JM, Kim YC, O E, Compans RW, Prausnitz MR, Kang SM. DNA vaccination in the skin using microneedles improves protection against influenza. Mol Ther. 2012;20(7):1472–80. 10.1038/mt.2012.69 22508490 PMC3392990

[21] Murphy KM. Janeway's Immunobiology 8th Ed: Garland Science; 2012.

[22] Davis S. Nasal vaccines. Adv Drug Del Rev. 2001;51(1):21–42. 10.1016/s0169-409x(01)00162-4 11516777

[23] Buonagurio DA, Bechert TM, Yang CF, Shutyak L, D'Arco GA, Kazachkov Y, et al Genetic stability of live, cold-adapted influenza virus components of the FluMist/CAIV-T vaccine throughout the manufacturing process. Vaccine. 2006;24(12):2151–60. 10.1016/j.vaccine.2005.11.007 . 16413951

[24] Carter NJ, Curran MP. Live Attenuated Influenza Vaccine (FluMist; Fluenz). Drugs. 2011;71(12):1591–622. 10.2165/11206860-000000000-00000 21861544

[25] Gill HS, Prausnitz MR. Coated microneedles for transdermal delivery. J Control Release. 2007;117(2):227–37. 10.1016/j.jconrel.2006.10.017 17169459 PMC1853346

[26] Quan FS, Kim YC, Yoo DG, Compans RW, Prausnitz MR, Kang SM. Stabilization of Influenza Vaccine Enhances Protection by Microneedle Delivery in the Mouse Skin. PLoS One. 2009;4(9). 10.1371/journal.pone.0007152 . PMC2745577 19779615

[27] Kim Y-C, Yoo D-G, Compans RW, Kang S-M, Prausnitz MR. Cross-protection by co-immunization with influenza hemagglutinin DNA and inactivated virus vaccine using coated microneedles. J Controlled Release. 2013. 10.1016/j.jconrel.2013.04.016 PMC3815987 23643528

[28] Tobita K, Sugiura A, Enomoto C, Furuyama M. Plaque assay and primary isolation of influenza A viruses in an established line of canine kidney cells (MDCK) in the presence of trypsin. Med Microbiol Immunol. 1975;162(1):9–14. 1214709 10.1007/BF02123572

[29] Ohmit SE, Thompson MG, Petrie JG, Thaker SN, Jackson ML, Belongia EA, et al. Influenza vaccine effectiveness in the 2011–2012 season: protection against each circulating virus and the effect of prior vaccination on estimates. Clin Infect Dis. 2013:cit736. 10.1093/cid/cit736 PMC4007111 24235265

[30] Pica N, Palese P. Toward a universal influenza virus vaccine: prospects and challenges. Annu Rev Med. 2013;64:189–202. 10.1146/annurev-med-120611-145115 . 23327522

[31] Subbarao K, Matsuoka Y. The prospects and challenges of universal vaccines for influenza. Trends Microbiol. 2013. 10.1016/j.tim.2013.04.003 PMC3700639 23685068

[32] Matsuo K, Hirobe S, Okada N, Nakagawa S. Frontiers of transcutaneous vaccination systems: Novel technologies and devices for vaccine delivery. Vaccine. 2013. 10.1016/j.vaccine.2013.03.022 PMC7125630 23523401

[33] Nestle FO, Di Meglio P, Qin J-Z, Nickoloff BJ. Skin immune sentinels in health and disease. Nature Reviews Immunology. 2009;9(10):679–91. 10.1038/nri2622 19763149 PMC2947825

[34] Pavot V, Rochereau N, Genin C, Verrier B, Paul S. New insights in mucosal vaccine development. Vaccine. 2012;30(2):142–54. 10.1016/j.vaccine.2011.11.003 22085556

[35] Sullivan SP, Koutsonanos DG, Del Pilar Martin M, Lee JW, Zarnitsyn V, Choi SO, et al Dissolving polymer microneedle patches for influenza vaccination. Nat Med. 2010;16(8):915–20. 10.1038/nm.2182 20639891 PMC2917494

[36] Takada A, Matsushita S, Ninomiya A, Kawaoka Y, Kida H. Intranasal immunization with formalin-inactivated virus vaccine induces a broad spectrum of heterosubtypic immunity against influenza A virus infection in mice. Vaccine. 2003;21(23):3212–8. 12804850 10.1016/s0264-410x(03)00234-2

[37] Wareing M, Tannock G. Live attenuated vaccines against influenza; an historical review. Vaccine. 2001;19(25):3320–30. 11348696 10.1016/s0264-410x(01)00045-7

[38] Kim YC, Quan FS, Yoo DG, Compans RW, Kang SM, Prausnitz MR. Enhanced memory responses to seasonal H1N1 influenza vaccination of the skin with the use of vaccine-coated microneedles. J Infect Dis. 2010;201(2):190–8. 10.1086/649228 20017632 PMC2798016

[39] Quan FS, Kim YC, Compans RW, Prausnitz MR, Kang SM. Dose sparing enabled by skin immunization with influenza virus-like particle vaccine using microneedles. J Control Release. 2010;147(3):326–32. 10.1016/j.jconrel.2010.07.125 20692307 PMC2975779

[40] Ichinohe T, Tamura S-i, Kawaguchi A, Ninomiya A, Imai M, Itamura S, et al Cross-protection against H5N1 influenza virus infection is afforded by intranasal inoculation with seasonal trivalent inactivated influenza vaccine. J Infect Dis. 2007;196(9):1313–20. 17922395 10.1086/521304 PMC7110255

[41] McGhee J, Czerkinsky C, Mestecky J. Mucosal vaccines: an overview. Mucosal Immunol. 1999;2:741–57.

[42] Yang P, Duan Y, Zhang P, Li Z, Wang C, Dong M, et al Multiple-clade H5N1 influenza split vaccine elicits broad cross protection against lethal influenza virus challenge in mice by intranasal vaccination. PLoS One. 2012;7(1):e30252 10.1371/journal.pone.0030252 22279575 PMC3261182

[43] Eyles JE, Williamson ED, Alpar HO. Immunological responses to nasal delivery of free and encapsulated tetanus toxoid: studies on the effect of vehicle volume. Int J Pharm. 1999;189(1):75–9. 10518687 10.1016/s0378-5173(99)00239-2

[44] Balmelli C, Roden R, Potts A, Schiller J, De Grandi P, Nardelli-Haefliger D. Nasal immunization of mice with human papillomavirus type 16 virus-like particles elicits neutralizing antibodies in mucosal secretions. J Virol. 1998;72(10):8220–9. 9733865 10.1128/jvi.72.10.8220-8229.1998 PMC110174

[45] Baz M, Samant M, Zekki H, Tribout-Jover P, Plante M, Lanteigne A-M, et al Effects of different adjuvants in the context of intramuscular and intranasal routes on humoral and cellular immune responses induced by detergent-split A/H3N2 influenza vaccines in mice. Clin Vaccine Immunol. 2012;19(2):209–18. 10.1128/CVI.05441-11 22190392 PMC3272927

[46] Richert LE, Servid AE, Harmsen AL, Rynda-Apple A, Han S, Wiley JA, et al A virus-like particle vaccine platform elicits heightened and hastened local lung mucosal antibody production after a single dose. Vaccine. 2012;30(24):3653–65. 10.1016/j.vaccine.2012.03.035 22465748 PMC3579574

[47] Li Y, Li J, Zhang X, Ding J, Mao S. Non-ionic surfactants as novel intranasal absorption enhancers: in vitro and in vivo characterization. Drug Deliv. 2014;(0):1–8. 10.3109/10717544.2014.971196 25347689

[48] Oh Y-K, Park J-S, Yoon H, Kim C-K. Enhanced mucosal and systemic immune responses to a vaginal vaccine coadministered with RANTES-expressing plasmid DNA using in situ-gelling mucoadhesive delivery system. Vaccine. 2003;21(17):1980–8. 12706687 10.1016/s0264-410x(02)00779-x

[49] Xu X, Shen Y, Wang W, Sun C, Li C, Xiong Y, et al Preparation and in vitro characterization of thermosensitive and mucoadhesive hydrogels for nasal delivery of phenylephrine hydrochloride. Eur J Pharm Biopharm. 2014;88(3):998–1004. 10.1016/j.ejpb.2014.08.015 25257714

[50] Chen X, Zhi F, Jia X, Zhang X, Ambardekar R, Meng Z, et al Enhanced brain targeting of curcumin by intranasal administration of a thermosensitive poloxamer hydrogel. J Pharm Pharmacol. 2013;65(6):807–16. 10.1111/jphp.12043 23647674

[51] Lehr C-M, Bouwstra JA, Schacht EH, Junginger HE. In vitro evaluation of mucoadhesive properties of chitosan and some other natural polymers. Int J Pharm. 1992;78(1):43–8.

[52] Ugwoke MI, Agu RU, Jorissen M, Augustijns P, Sciot R, Verbeke N, et al Toxicological investigations of the effects carboxymethylcellulose on ciliary beat frequency of human nasal epithelial cells in primary suspension culture and in vivo on rabbit nasal mucosa. Int J Pharm. 2000;205(1):43–51. 11000541 10.1016/s0378-5173(00)00484-1

[53] Roy S, Pal K, Anis A, Pramanik K, Prabhakar B. Polymers in mucoadhesive drug-delivery systems: a brief note. Designed monomers and polymers. 2009;12(6):483–95.

[54] Madsen F, Eberth K, Smart JD. A rheological examination of the mucoadhesive/mucus interaction: the effect of mucoadhesive type and concentration. J Controlled Release. 1998;50(1):167–78. 10.1016/s0168-3659(97)00138-7 9685883

[55] Prausnitz MR, Mikszta JA, Cormier M, Andrianov AK. Microneedle-based vaccines Vaccines for Pandemic Influenza: Springer; 2009 p. 369–93. 10.1007/978-3-540-92165-3_18 PMC2904604 19768415

[56] Song JY, Cheong HJ, Noh JY, Yang TU, Seo YB, Hong K-W, et al Long-term immunogenicity of the influenza vaccine at reduced intradermal and full intramuscular doses among healthy young adults. Clinical and experimental vaccine research. 2013;2(2):115–9. 10.7774/cevr.2013.2.2.115 23858402 PMC3710919

[57] Budimir N, Haan A, Meijerhof T, Gostick E, Price DA, Huckriede A, et al Heterosubtypic cross‐protection induced by whole inactivated influenza virus vaccine in mice: influence of the route of vaccine administration. Influenza Other Respi Viruses. 2013;7(6):1202–9. 10.1111/irv.12142 PMC4112805 24102979

