# Supplementary figures and images for: Microneedle Vaccination Elicits Superior Protection and Antibody Response over Intranasal Vaccination against Swine-Origin Influenza A (H1N1) in Mice
Source: PLoS One. 2015 Jun 18;10(6):e0130684. doi: 10.1371/journal.pone.0130684 (PMC4472750; doi:10.1371/journal.pone.0130684)

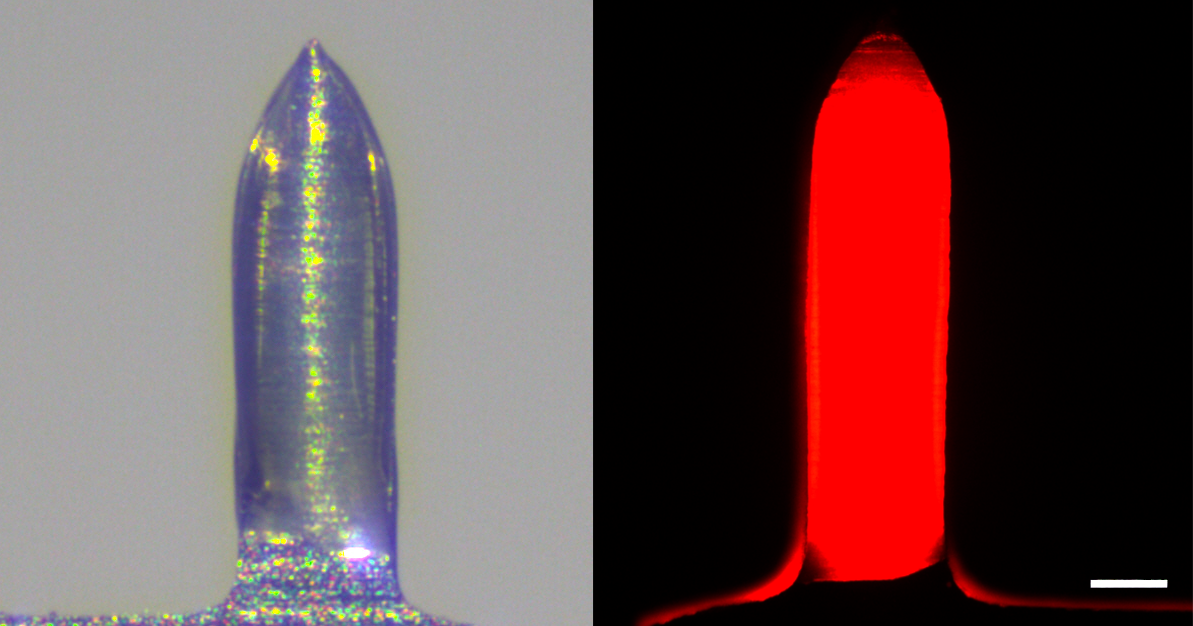

Supplement: S1 Fig — In-line stainless steel microneedle array was coated with coating solution plus BSA or Sulforhodamine B and observed by optical microscopy or fluorescence microscopy, respectively. Scale bar = 100 μm. (TIF) [file pone.0130684.s001.tif]

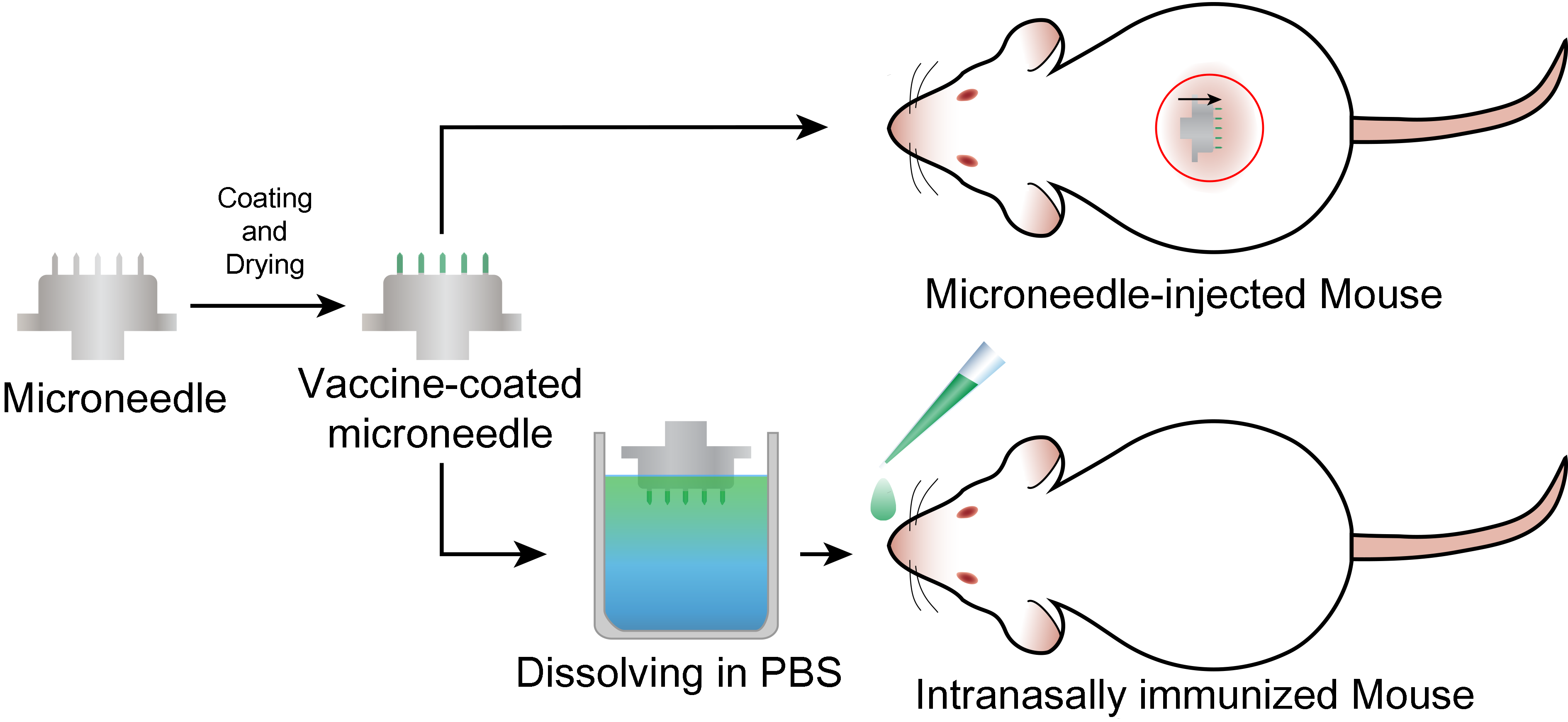

Supplement: S2 Fig — One group of mice were immunized with a vaccine solution, consisting of 1 μg of inactivated swine H1N1 virus vaccine constituted in a coating solution, by injection of the solution into nostrils. In the other group of mice, vaccine-coated MN were inserted into the back skin of mice, which were coated with the same dosage of the vaccine as in IN vaccination. (TIF) [file pone.0130684.s002.tif]
